# Downregulation of SAV1 plays a role in pathogenesis of high-grade clear cell renal cell carcinoma

**DOI:** 10.1186/1471-2407-11-523

**Published:** 2011-12-20

**Authors:** Keiko Matsuura, Chisato Nakada, Mizuho Mashio, Takahiro Narimatsu, Taichiro Yoshimoto, Masato Tanigawa, Yoshiyuki Tsukamoto, Naoki Hijiya, Ichiro Takeuchi, Takeo Nomura, Fuminori Sato, Hiromitsu Mimata, Masao Seto, Masatsugu Moriyama

**Affiliations:** 1 Department of Molecular Pathology, Oita University, Oita, Japan; 2 Department of Urology, Faculty of Medicine, Oita University, Oita, Japan; 3 Division of Biomolecular Medicine and Medical Imaging, Oita University, Oita, Japan; 4 Department of Scientific and Engineering Simulation, Graduate School of Engineering, Nagoya Institute of Technology, Nagoya, Japan; 5 Division of Molecular Medicine, Aichi Cancer Center, Nagoya, Japan; 6 Department of Pathology and Diagnostic Pathology, Graduate School of Medicine, University of Tokyo, Tokyo, Japan; 7 Department of Molecular Pathology, Faculty of Medicine, Oita University, Hasama-machi, Yufu-city, Oita 879-5593, Japan

**Keywords:** Clear cell renal cell carcinoma, SAV1, Hippo pathway

## Abstract

**Background:**

Clinical outcome of patients with high-grade ccRCC (clear cell renal cell carcinoma) remains still poor despite recent advances in treatment strategies. Molecular mechanism of pathogenesis in developing high-grade ccRCC must be clarified. In the present study, we found that SAV1 was significantly downregulated with copy number loss in high-grade ccRCCs. Therefore, we investigated the SAV1 function on cell proliferation and apoptosis in vitro. Furthermore, we attempted to clarify the downstream signaling which is regulated by SAV1.

**Methods:**

We performed array CGH and gene expression analysis of 8 RCC cell lines (786-O, 769-P, KMRC-1, KMRC-2, KMRC-3, KMRC-20, TUHR4TKB, and Caki-2), and expression level of mRNA was confirmed by quantitative RT-PCR (qRT-PCR) analysis. We next re-expressed SAV1 in 786-O cells, and analyzed its colony-forming activity. Then, we transfected siRNAs of SAV1 into the kidney epithelial cell line HK2 and renal proximal tubule epithelial cells (RPTECs), and analyzed their proliferation and apoptosis. Furthermore, the activity of YAP1, which is a downstream molecule of SAV1, was evaluated by western blot analysis, reporter assay and immunohistochemical analysis.

**Results:**

We found that SAV1, a component of the Hippo pathway, is frequently downregulated in high-grade ccRCC. SAV1 is located on chromosome 14q22.1, where copy number loss had been observed in 7 of 12 high-grade ccRCCs in our previous study, suggesting that gene copy number loss is responsible for the downregulation of SAV1. Colony-forming activity by 786-O cells, which show homozygous loss of SAV1, was significantly reduced when SAV1 was re-introduced exogenously. Knockdown of SAV1 promoted proliferation of HK2 and RPTEC. Although the phosphorylation level of YAP1 was low in 786-O cells, it was elevated in SAV1-transduced 786-O cells. Furthermore, the transcriptional activity of the YAP1 and TEAD3 complex was inhibited in SAV1-transduced 786-O cells. Immunohistochemistry frequently demonstrated nuclear localization of YAP1 in ccRCC cases with SAV1 downregulation, and it was preferentially detected in high-grade ccRCC.

**Conclusions:**

Taken together, downregulation of SAV1 and the consequent YAP1 activation are involved in the pathogenesis of high-grade ccRCC. It is an attractive hypothesis that Hippo signaling could be candidates for new therapeutic target.

## Background

Renal cell carcinoma (RCC) is histopathologically subdivided into various categories, of which clear cell renal cell carcinoma (ccRCC) is the most common subtype, accounting for 70-80% of all RCCs [1]. Fuhrman's nuclear grading system is used as a reliable prognostic indicator for ccRCCs [2], and it is widely accepted that the clinical outcome of patients with high-grade ccRCC remains poor despite recent advances in treatment strategies [3-6]. Therefore, it is important to clarify the pathogenesis of high-grade ccRCC in order to develop new treatments for improving the prognosis of affected patients. However, differences in the molecular mechanisms of pathogenesis between low-grade and high-grade ccRCCs remain to be determined.

We have previously reported the presence of chromosomal copy number aberrations (CNAs) in ccRCC determined using array-based CGH analysis [7]. In our previous study, 14q loss was observed in 7 of 12 high-grade ccRCCs, but in only 1 of 10 low-grade ccRCCs, suggesting that 14q loss is important for the development of the former. Furthermore, we had previously found that genes located at 14q with copy number loss tended to be downregulated, suggesting that copy number loss at 14q in high-grade ccRCC is responsible for the downregulation of genes located in this region, and that putative tumor suppressor genes may be present at this chromosomal locus [7]. However, the genes concerned at 14q have not yet been identified.

In the present study, we attempted to identify genes that are downregulated as a result of copy number loss at 14q by using ccRCC cell lines in addition to clinical samples. We found that the SAV1 gene, a human homolog of *salvador*, which is known to be a tumor suppressor in *Drosophila *[8], was significantly downregulated in ccRCCs with 14q loss. Further analysis of SAV1 function revealed that it is a putative tumor suppressor gene in high-grade ccRCCs.

## Methods

### Cell culture

The renal cell carcinoma cell lines 786-O (#CRL-1932), 769-P (#CRL-1933) and Caki-2 (#HTB-47), and the human cell line HK2 (#CRL-1427), were purchased from the American Type Culture Collection (ATCC) (Rockville, MD). KMRC-1, KMRC-2, KMRC-3, and KMRC-20 were purchased from JCRB (Osaka, Japan), and TUHR4TKB was provided by RIKEN BRC through the National Bio-Resource Project of MEXT, Japan. RPTEC was purchased from Lonza Walkersville Inc. (Walkersville, MD). All cell lines were maintained in accordance with the supplier's instructions.

### Tissue samples and histopathological examination

Primary ccRCCs were surgically resected at Oita University Hospital, and diagnosed histopathologically as described previously [7]. Information on the other 98 patients is summarized in Additional file 1: Table S1. Use of the tissue samples for all experiments was approved by all the patients and by Oita University Ethics Committee (Approval No. P-05-05).

### Immunohistochemistry

Immunohistochemistry of paraffin-embedded RCC tissue sections was performed in a similar way to that used in our previous work [9]. Anti-SAV1 antibody (clone 3B320; Abnova, Taipei, Taiwan) and anti-YAP antibody (Cell Signaling Technology, Danvers) were used as the primary antibodies.

### Quantitative RT-PCR

Quantitative RT-PCR was performed with a Universal probe library (Roche Diagnostics, Mannheim, Germany) and LightCycler 480 probe master (Roche Diagnostics) by the Taqman method as described previously [10]. Levels of messenger RNA expression relative to KPNA6 were obtained from a standard curve. We used KPNA6 as a control in this study because KPNA6 expression was not statistically different among the ccRCC and non-neoplastic tissues, although expression levels of known markers such as beta-2-microgloblin and glyceraldehydes 3-phosphate dehydrogenase (GAPDH) were variable across the samples [11].

### Lentiviral vector production and in vitro transduction

The full-length SAV1 cDNA was obtained from NBRC (NITE Biological Resource Center, Chiba, Japan), cloned into pLenti7.3/V5-DEST (Invitrogen, Carlsbad, CA, USA) using the Gateway system^™ ^in accordance with the manufacturer's instructions, and used to generate the vector SAV1-pLenti7.3/V5-DEST (pLenti-SAV1). Transient transfections of the pLenti7.3/V5-DEST empty vector and pLenti-SAV1 with ViraPower Packaging Mix (Invitrogen) into HEK293T cells were performed using Lipofectamine2000 (Invitrogen) in accordance with the manufacturer's instructions. After 48 h, the viral supernatants were collected, filtered, and enriched by ultracentrifugation. Subsequently, transduction of target cells was performed at an optimized multiplicity of infection (MOI) of 5 with Polybrene at a final concentration of 6.0 μg/ml. To identify stable clones of SAV1-re-expressing cells, the full-length SAV1 cDNA was cloned into pLenti6.3/V5-DEST (Invitrogen). Lentivirus expressing SAV1 was generated as described above. 786-O cells were transduced with SAV1-pLenti6.3/V5-DEST or the pLenti6.3/V5-DEST vector, respectively. After transduction and selection with 0.5 μg/ml blasticidin (Invitrogen), two independent clones for SAV1 were obtained, and designated as SAV1-1 and SAV1-2. Control cells were also generated using the pLenti6.3/V5-DEST empty vector.

### Colony formation assay

One day after transduction of 786-O or 769-P cells with lentivirus expressing SAV1, one fiftieth of the cells were reseeded on 100-mm dishes and cultured. When colonies became visible, they were stained with Giemsa for 30 min, rinsed with water, and counted.

### siRNA transfection

Cells were transfected with Stealth^™ ^RNAi oligonucleotide or the Stealth^™ ^RNAi Negative Control Duplex with the corresponding GC content (Invitrogen) at a final concentration of 10 nM using Lipofectamine RNA-MAX (Invitrogen) in accordance with the manufacturer's instructions.

### Proliferation assay

After transfection of siRNAs in a 96-well plate, MTS assay was carried out using the CellTiter 96^R^AQ_ueous _One Solution Cell Proliferation Assay Kit (Promega, Madison, WI, USA), and the optical density was measured at 492 nm using a fluorescence reader (Tecan SpectraFluor) (Tecan, Crailsheim, Germany). In addition, BrdU incorporation was performed using a Cell Proliferation ELISA Kit (Roche Applied Science, Mannheim, Germany) in accordance with the manufacturer's protocols.

### Apoptosis assay

Cells were transfected with siRNA in a 96-well plate and cultured for 72 h. Nuclear morphology was then evaluated for apoptosis. DNA fragmentation was detected in the 96-well format using Cell Death Detection ELISA plus (Roche Applied Science) in accordance with the manufacturer's instructions. The activity of caspase 3 and caspase 7 was detected in the 96-well format by using the caspase-Glo 3/7 Assay (Promega) according to the manufacturer's instructions.

### Western blot analysis

Western blot analysis was performed similarly to that in our previous study [12]. In each analysis, 10 μg of cell lysate was used. The primary antibodies employed were: anti-human GAPDH antibody (Sigma-Aldrich), anti-SAV1 antibody (Cell Signaling Technology), anti-phosphorylated YAP antibody (Ser127; Cell Signaling Technology), and anti-YAP antibody (Santa Cruz Biotechnology). Detection was performed with ECL Western Blotting Detection Reagents (Amersham Biosciences, Piscataway, NJ, USA) in accordance with the manufacturer's instructions.

### Dual luciferase assay

For the luciferase reporter assay, stable clones of 786-O cells (5 × 10^4^) were seeded in 6-well plates, and then co-transfected with 0.5 μg of pGL4.35 (9xUAS Gal4) and GAL4-TEADs, respectively, using Lipofectamine Plus (Invitrogen). At 48 h after transfection, the cells were lysed and luciferase activity was assayed using a Dual-luciferase Reporter Assay kit (Promega) in accordance with the manufacturer's instructions. All luciferase activities were normalized to the *Renilla *luciferase reporter pRL-CMV plasmid (Promega).

### Statistical analysis

Quantitative RT-PCR data were analyzed statistically by the Mann-Whitney *U*-test. The two-sided Student's *t *test and Fisher's exact test was used to analyze differences in experimental data obtained from the cell lines. Differences at *P *< 0.05 were considered statistically significant.

Additional methods are described in Supplementary methods (Additional file 2: Methods S1)

## Results

### SAV1 is downregulated in RCCs with copy number loss at 14q22.1

We performed array CGH analysis and gene expression analysis of 8 RCC cell lines (786-O, 769-P, KMRC-1, KMRC-2, KMRC-3, KMRC-20, TUHR4TKB, and Caki-2) in order to identify downregulated genes located in the region of 14q loss (Additional file 3: Figure S1a, [7]). All the data obtained in the array CGH and expression microarray analysis are available at DDBJ via CIBEX (http://cibex.nig.ac.jp/index.jsp), under accession numbers CBX97 for array CGH, and CBX96 for expression-microarray. Copy number loss at 14q was detected in all the cell lines analyzed. Detailed analysis of the region of copy number loss at 14q revealed two different homozygous deletions at 14q in 3 (786-O, 769-P, KMRC-1) of the 8 cell lines.

**Figure 1:**
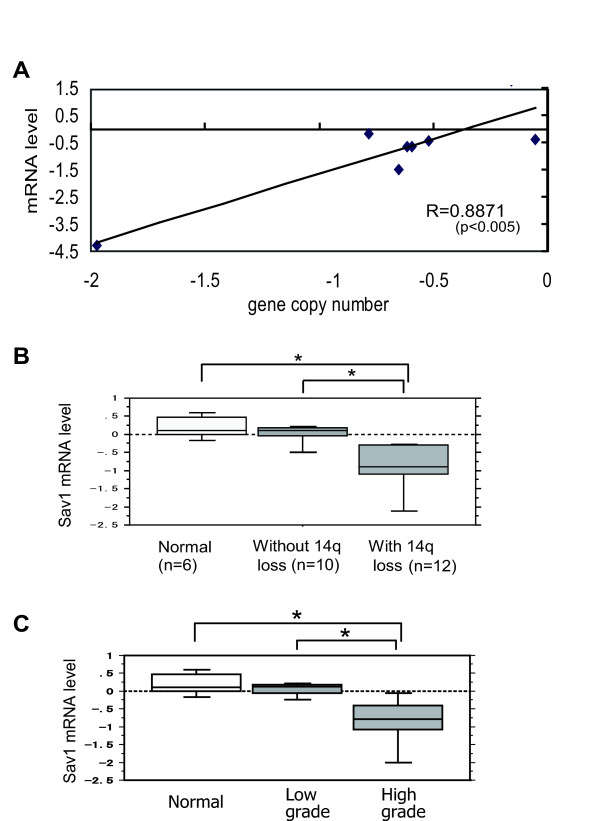
**SAV1 is downregulated in RCC with copy number loss at 14q22.1**. **a **Positive correlation of copy number with the expression ratio (R = 0.8871) of the SAV1 gene in RCC cell lines (786-O, 769-P, KMRC-1, KMRC-2, KMRC-3, KMRC-20, TUHR4TKB, and Caki-2). Log_2 _ratio of the normalized relative signal intensity of SAV1 by microarray (Y-axis) and log_2 _ratio of the gene copy number (X-axis) are plotted for each cell line. R, Pearson correlation coefficient. *p*-values were calculated by *t *test. **b **Correlation of the expression level with copy number loss of SAV1 mRNA at 14q. Quantitative RT-PCR was performed for ccRCC cases that had been analyzed by array CGH in our previous study [7]. On the basis of gene copy number ratio, ccRCC cases were divided into two groups [cases without 14q loss; log_2 _ratio -0.14 to 0.15 (n = 10), and cases with 14q loss; log_2 _ratio < -0.14 (n = 12)] and analyzed for their expression of SAV1. The box plots show the log_2 _ratio of SAV1 mRNA normalized by the median value for 6 samples of normal kidney (Normal). SAV1 mRNA levels are significantly lower in cases with 14q loss than in those without, and normal kidney (*p *< 0.005). The Y-axis displays the expression level (log_2_). *P *values were calculated by the Mann-Whitney *U*-test. *p < 0.005. Vertical lines in each column indicate mean ratio, and bars show s.d.. **c **Correlation of the expression level of SAV1 mRNA with tumor grade. The box plots show log_2 _ratio of SAV1 mRNA normalized by the median value for 6 samples of normal kidney. SAV1 mRNA levels are significantly lower in high-grade ccRCC tissue than in low-grade ccRCC and normal kidney (*p *< 0.005).

In 786-O, a homozygous loss was detected at 14q22.1 (Additional file 3: Figure S1b), and this encompassed three genes: *SPG*, *SAV1*, and *MAP4K5 *(Additional file 3: Figure S1c). On the other hand, in 769-P and KMRC-1 cells, a homozygous loss was detected at 14q23.1, where only the *HIF-1a *gene is located. Next, using array CGH data and transcriptome data for these 8 cell lines, we analyzed whether the expression levels of the *SPG*, *SAV1*, *MAP4K5*, and *HIF-1a *genes were correlated with their gene copy number status. As shown in Figure 1a, the SAV1 gene showed a positive correlation between the copy number change and the expression ratio (R = 0.8871; *p *< 0.005) (Figure 1a). In contrast, copy number loss was not significantly correlated with the level of mRNA expression for the *SPG*, *MAP4K *and *HIF-1a *genes (Additional file 4: Figure S2), suggesting that SAV1 downregulation is correlated with gene copy number loss at 14q in these cell lines. To confirm this trend in ccRCC cases, quantitative RT-PCR (qRT-PCR) analysis of ccRCC samples was performed, and this revealed that the levels of SAV1 mRNA in cases showing 14q loss (n = 10) were significantly lower than those without 14q loss (n = 12) (*p *< 0.005), although they were similar between cases without 14q loss and normal kidney (Figure 1b). Furthermore, qRT-PCR analysis of ccRCC samples revealed that the levels of SAV1 mRNA in high-grade CCCs (n = 7) were significantly lower than those in low-grade CCCs (n = 8) and normal kidney (n = 5) (*p *< 0.005) (Figure 1c). In addition, protein level of SAV1 in ccRCC cases was evaluated by immunohistochemistry. SAV1 protein was detected in proximal renal tubules, podocytes, and endothelial cells in normal tissues (Additional file 5: Figure S3). The specificity of SAV1 antibody was confirmed by immunocytochemistry of 786-O cells transduced with lentivirus expressing SAV1 or control virus (Additional file 6: Figure S4). In tumor cells, SAV1 downregulation at the protein level was observed in 64 of the 98 ccRCC cases, and it was correlated with tumor grade (Table 1). These findings suggest that SAV1 is downregulated in RCC cell lines as well as ccRCC samples and that its downregulation occurs preferentially in high-grade ccRCCs. To evaluate the SAV1 gene mutation, we performed PCR for 6 ccRCC cases with SAV1 downregulation using 5 pairs of primers within open reading frame and we could not detect any mutations (data not shown), suggesting that gene mutation does not contribute to SAV1 inactivation in RCC.

**Table 1:** Impact of SAV1 protein expression and nuclear grade in ccRCC

		SAV1 immunoreactivity		
	
		Positive	weak or negative	Total
nuclear	low	16	17	33
grade	high	18	47	65

	total	34	64	98
		Fisher's exact test		*p *= 0.0469

### SAV1 inhibits colony formation and cell proliferation

**Figure 2:**
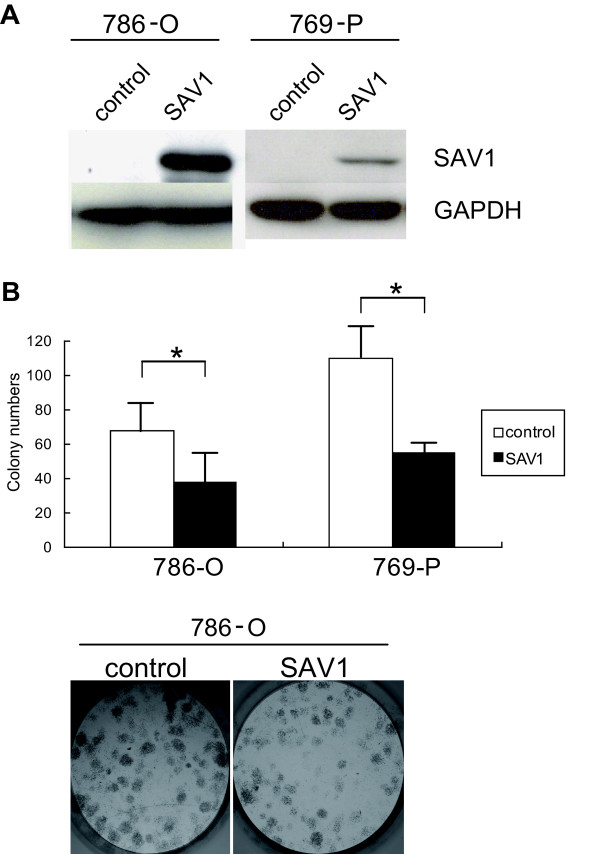
**Re-expression of SAV1 inhibits cell proliferation and colony formation**. **a **SAV1 protein is expressed in 786-O and 769-P cells transduced with SAV1. Total cell lysates were subjected to Western blot analysis using anti-SAV1 antibody and anti-GAPDH antibody, respectively. SAV1 protein was detected in SAV1-transduced 786-O and 769-P cells (SAV1), but not in cells transduced with pLenti7.3/V5 empty vector (control). **b **Re-expression of SAV1 inhibits colony-formation in 786-O and 769-P cells. Upper column; colony-forming activities of 786-O and 769-P cells transduced with control vector or pLenti-SAV1. Colony numbers were significantly reduced in 786-O and 769-P cells transduced with pLenti-SAV1 (SAV1) compared with those transduced with pLenti7.3/V5 (control). Experiments were performed in triplicate. Lower column; A representative result of colony formation assay using 786-O cells transduced with the control vector and those transduced with SAV1.

We next re-expressed SAV1 in 786-O cells, in which SAV1 is homozygously deleted, by transducing them with SAV1 (Figure 2a), and analyzed their colony-forming activity. As shown in Figure 2b, colony-forming activity was significantly reduced in SAV1-transduced 786-O cells. Similar observations were obtained for 769-P cells (Figure 2a, b). Furthermore, this trend was also confirmed by MTS assay (Additional file 7: Figure S5).

**Figure 3:**
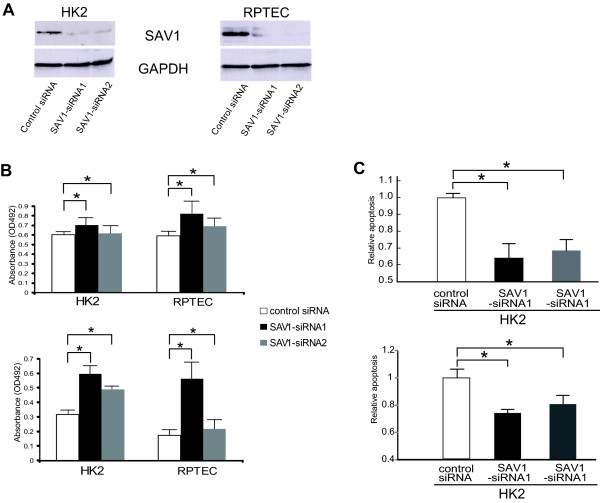
**Knockdown of SAV1 induces cell proliferation and inhibits apoptosis**. **a **Expression of SAV1 protein was suppressed in HK2 cells and RPTEC transfected with specific siRNAs. Two independent Stealth^™ ^RNAi oligonucleotides for SAV1 (SAV1-siRNA1 and SAV1-siRNA2), or the Stealth^™ ^RNAi Negative Control Duplex siRNAs (control-siRNA) were transfected into HK2 and RPTEC cells. After 72 h, protein was extracted and analyzed by Western blotting with anti-SAV1 antibody and anti-GAPDH antibody. SAV1 was markedly downregulated in HK2 and RPTEC cells transfected with SAV1-siRNA1 and SAV1-siRNA2. **b **Knockdown of SAV1 induces proliferation of HK2 cells and RPTEC. Upper column; MTS assay at 72 h after transfection. Y axis indicates the absorbance at 492 nm for MTS assay. Experiments were performed in triplicate, and bars indicate s.d.. * *p *< 0.05; Student's *t *test. Lower column; BrdU incorporation was estimated as the absorbance of cells transfected with SAV1-siRNA1 and SAV1-siRNA2, and those transfected with control siRNA. Experiments were performed in triplicate, and bars indicate s.d.. * *p *< 0.05; Student's *t *test. MTS assay as well as BrdU incorporation demonstrated that proliferation of HK2 cells and RPTEC transfected with SAV1-siRNA1 and SAV1-siRNA2 was significantly increased relative to cells transfected with control siRNA. **c **Apoptotic activity after transfection with SAV1 siRNAs was significantly inhibited. Upper column; HK2 cells transfected with control siRNA, SAV1-siRNA1 and SAV1-siRNA2 were incubated for 72 h and then analyzed using a cell death ELISA kit. Y axis indicates the relative ratio of the absorbance at 492 nm for control cells set at 1.0. The results are shown as means ± s.d. of three independent experiments. * *p *< 0.001; Student's *t *test. Lower column; HK2 cells transfected with control siRNA, SAV1-siRNA1 and SAV1-siRNA2 were incubated for 72 h and then analyzed using a Caspase-Glo 3/7 Assay kit. Y axis indicates the relative ratio of the fluorescent signal for control cells set at 1.0. The results are shown as means ± s.d. of three independent experiments. *p < 0.001; Student's *t *test.

We next transfected small interfering RNAs (siRNAs), designed to target distinct sites of SAV1 mRNA, into the kidney epithelial cell line HK2 and renal proximal tubule epithelial cells (RPTECs), and analyzed their proliferation (Figure 3a). We found that cell proliferation was promoted in HK2 and RPTECs transfected with SAV1-siRNA (Figure 3b), whereas the cell cycle of HK2 cells was not affected by SAV1-siRNA transfection (Additional file 8: Figure S6). However, the apoptotic activity of HK2 cells transfected with SAV1-siRNA was significantly reduced in cells transfected with SAV1-siRNA in comparison with those transfected with the control (Figure 3c), suggesting that SAV1 downregulation inhibits apoptosis in renal tubule cells.

### Downregulation of SAV1 activates YAP

**Figure 4:**
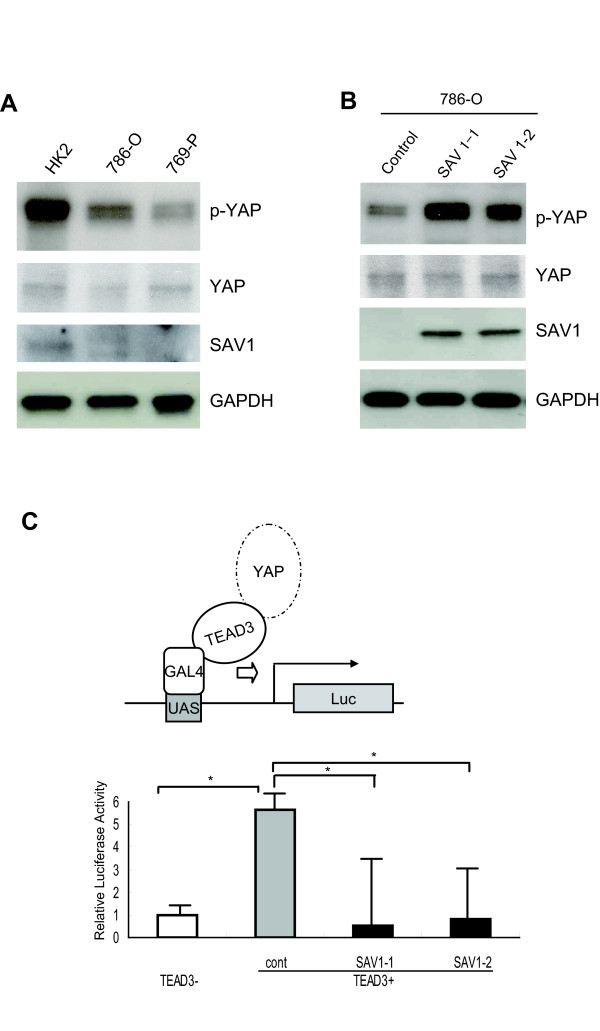
**Downregulation of SAV1 activates YAP1**. **a **Phosphorylation of YAP1 was decreased in RCC cell lines. Phosphorylation of YAP1 in HK2, 786-O and 769-P cells was analyzed by Western blot analysis with anti-phospho-YAP antibody, anti-YAP antibody, and anti-GAPDH antibody, respectively. Protein level of SAV1 in each cell line was demonstrated with anti-SAV1 antibody. SAV1 was detectable only in HK2 cells. **b **Phosphorylated YAP1 is increased in SAV1-transduced 786-O cells. 786-O cells were transduced with pLenti6.3/V5-DEST empty vector or SAV1-pLenti6.3/V5-DEST, and one clone of control cells (designated as the control) and two independent clones of SAV1-re-expressing cells (designated as SAV1-1 and SAV1-2) were established. Western blotting was performed with anti-phospho-YAP antibody, anti-YAP antibody, anti-SAV1 antibody and anti-GAPDH antibody, respectively. **c **SAV1 suppresses transcriptional activity of YAP1-TEAD3. SAV1-1, SAV1-2 and control cells were transfected with the Gal4-TEAD3 plasmid vector that expresses TEAD3 fused to GAL4 (kindly provided by Dr. B. Zhao [19]), Gal4-9x UAS luciferase reporter (pGL4.31), and pRL-CMV. Firefly luciferase activity was normalized to Renilla luciferase activity. Normalized luciferase activity in control cells untransfected with TEAD3 was set at 1. In SAV1-1 and SAV1-2 cells, the luciferase activity was significantly decreased. Experiments were performed in triplicate. The dual luciferase data are shown as mean ± SD. *p < 0.005 *; Student's *t *test.

It has been reported that SAV1 may function as a component of the Hippo signaling pathway, and the Hippo pathway targets the transcriptional co-activator YAP1 [13-17]. YAP1 is phosphorylated by LATS1/2, and the phosphorylated YAP1 is then kept localized in the cytoplasm. On the other hand, when Hippo signaling is inhibited, unphosphorylated YAP1 accumulates in the nucleus, where it forms a transcriptionally active complex with the transcription factor, TEAD [18,19]. To determine whether Hippo signaling is actually inhibited in RCCs in which SAV1 is frequently downregulated, we analyzed the phosphorylation status of YAP1 in 786-O and 769-P cells in which SAV1 are downregulated and HK2 cells in which SAV1 is expressed at a comparable level (Figure 4a). As shown in Figure 4a, phosphorylated YAP1 was significantly decreased in 786-O and 769-P cells, relative to HK2 cells. Furthermore, YAP1 phosphorylation was enhanced when SAV1 was re-expressed in 786-O cells (Figure 4b).

It has been reported that unphosphorylated YAP1 binds to members of the TEAD family, including TEAD1, 2, 3 and 4, and plays a functional role as a transcriptional co-activator [17]. Therefore, we next examined whether SAV1 affects the transcriptional activity of the YAP1-TEAD complex in RCCs. SAV1-transduced 786-O cells were transfected with expression plasmids encoding a Gal4 DNA binding domain fused to TEAD1, TEAD2, and TEAD3 (Gal4-TEADs), a Gal4-9x UAS luciferase reporter (pGL4.31) and pRL-CMV (Figure 4c) (Additional file 9: Figure S7). In control cells, co-transfection of the Gal4-TEAD3 and Gal4-9x UAS luciferase reporter led to an increase in luciferase activity. On the other hand, in SAV1-transduced 786-O cells, the luciferase activity eventually decreased, suggesting that, in RCCs with suppression of Hippo signaling, YAP1 activity is significantly elevated (Figure 4c).

**Figure 5:**
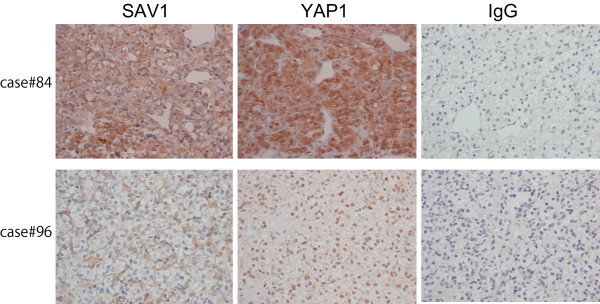
**Nuclear localization of YAP1 is frequently detectable in ccRCCs with SAV1 downregulation**. Immunohistochemistry of ccRCC tissues using anti-SAV1 antibody and anti-YAP1 antibody (×400). Representative results are shown. YAP1 tends to be localized in the nucleus of tumor cells in cases showing downregulation of SAV1 (case #96), whereas it tends to be localized mainly in the cytoplasm of tumor cells without downregulation of SAV1 (case #84). The cytoplasm of endothelial cells is positively immunostained. No positive staining is detectable in both cases using normal mouse IgG as the primary antibody (IgG).

To further confirm whether intracellular localization of YAP1 is under the control of Hippo signaling, we immunohistochemically analyzed the 98 cases of ccRCC for expression of YAP1. We compared the intracellular localization of YAP1 between ccRCCs showing downregulation of SAV1 and those without SAV1 downregulation. As shown in Figure 5 and Table 2, nuclear localization of YAP1 was more frequently detectable in ccRCCs with SAV1 downregulation than in those without, suggesting that SAV1 downregulation leads to nuclear translocation of YAP1. When we evaluated the correlation between nuclear localization of YAP1 and tumor grade, nuclear localization of YAP1 was preferentially detected in high-grade (Table 3), suggesting that the suppression of Hippo signaling through SAV1 downregulation tends to occur in high-grade ccRCCs.

**Table 2:** Immunohistochemical staining of SAV1 and YAP1 protein in ccRCC

		SAV1 immunoreactivity		
		
		positive	weak or negative	Total
YAP1	Cytoplasmic pattern*	28	9	37
	Nuclear pattern**	7	31	38
	
	total	35	40	75
		Fisher's exact test		*p *< 0.001

*Cytoplasmic pattern; YAP1 is mainly localized in cytoplasm of tumor

**Nuclear pattern; YAP1 is mainly localized in nucleus of tumor cells

**Table 3:** Impact of subcellular localization of YAP1 protein and nuclear grade in ccRCC

		nuclear grade		
		
		low	high	total
YAP1	Cytoplasmic pattern*	15	22	37
	Nuclear pattern**	7	31	38

	total	22	53	75
		Fisher's exact test		*p *= 0.0445

*Cytoplasmic pattern; YAP1 is mainly localized in cytoplasm of tumor

**Nuclear pattern; YAP1 is mainly localized in nucleus of tumor cells

## Discussion

The Hippo pathway is known to control organ size both in *Drosophila *and mammalian tissues, and many studies of the tumor suppressor function of the Hippo pathway have been reported [13,17,20-22]. In the present study, we found that SAV1 downregulation caused by 14q loss conferred a survival and growth advantage on RCCs. It has already been reported that the SAV1 gene is homozygously deleted in two RCC cell lines [8]. However, it has not been clarified whether the same abnormalities actually occur in patients with RCCs. Therefore, our present data appear to provide strong support for the hypothesis that the SAV1 gene may be a potential tumor suppressor of RCC. Furthermore, SAV1 has been reported to act as a tumor suppressor in cancers other than RCC. For example, two groups have recently reported that conditional knockout mice, in which the SAV1 gene was deleted only in the liver, developed hepatocellular carcinoma (HCC) [23,24], suggesting that deletion of the SAV1 gene is responsible for the development of such carcinomas. Therefore, we propose that the SAV1 gene is also involved in tumorigenesis of ccRCC.

In the present study, we found that knockdown of SAV1 induced significant inhibition of apoptosis in immortalized kidney cell line, HK2. It has previously been reported that apoptosis was inhibited in sav-mutated eye imaginal disc of *Drosophilla *[8]. Furthermore, in SAV1 gene-disrupted mice, apoptotic keratinocytes of epidermis was found to be significantly reduced compared with wild type mice [25]. Thus, on the basis of our present data in addition to the previous reports, it is suggested that downregulation of SAV1 may play an important role in inhibiting apoptosis in RCC.

On the other hand, in the present study, the increase of cell proliferation by SAV1 downregulation was relatively modest. With respect to interpretation of this result, we cannot rule out the possibility that the data indicating the increase of cell proliferation shown by MTS assay and BrdU-incorporation study may be affected by the significant inhibition of apoptosis caused by SAV1 knockdown. It has been reported that BrdU incorporation was significantly increased in liver of SAV1 gene-disrupted mice [24]. However, another group has reported that the increase of proliferation of embryonic keratinocytes isolated from SAV1-null mice was comparable to that of wild type [25]. Further study is needed to clarify whether SAV1 downregulation is really responsible for cell proliferation in RCC.

We have previously reported that loss of 14q was observed more frequently in cases of high-grade ccRCC than in low-grade ccRCC [7]. Since it has been reported that the prognosis of patients with high-grade ccRCCs is markedly poor, loss of 14q is suggested to be involved in the malignant phenotype of high-grade ccRCC [7]. In the present study, SAV1 downregulation was confined to high-grade ccRCCs with copy number loss at 14q encompassing 14q22.1. Although we cannot rule out the possibility that tumor suppressor genes other than SAV1 may exist at 14q, we speculate that suppression of the Hippo pathway caused by downregulation of SAV1 is involved in the development of high-grade ccRCCs.

It has been reported that YAP1 is a transcriptional regulator and acts as a oncoprotein in various carcinomas [26-30]. Indeed, when YAP1 is hypophosphorylated due to suppression of the Hippo signaling pathway including SAV1, it translocates from the cytoplasm to the nucleus and binds to various transcription factors [16,17,25]. An experiment using conditional knockout mice in which the SAV1 gene was deleted only in the liver and YAP1 was specifically activated in oval cells has clearly shown that SAV1 is required for inactivation of YAP1 [23]. In the present study, we found that phosphorylation of YAP1 was suppressed in SAV1-deficient RCC cell lines and that phosphorylation of YAP1 was enhanced in 786-O cells when SAV1 was re-expressed. Furthermore, YAP1 tended to translocate to the nucleus in SAV1-downregulated ccRCCs as well as in high-grade ccRCCs. These findings suggest that activation of YAP1 due to downregulation of SAV1 is involved in the pathogenesis of high-grade ccRCCs.

Recently, Zhao et al. reported that the transcription factor TEAD binds to YAP, and that the resulting YAP1-TEAD complex is required for YAP-induced cell growth and epithelial mesenchymal transition (EMT) [17]. We found that the transcriptional activity of TEAD-YAP was repressed when SAV1 was re-expressed in SAV1-deficient 786-O cells, suggesting that TEAD-YAP may play functional roles in the regulation of cell proliferation in RCC. However, at this stage, the transcriptional targets of the TEAD-YAP transcription factor complex in RCCs are still unclear. In order to clarify the molecular mechanisms involved in the development of high-grade RCCs, it will be necessary to identify the transcriptional target genes of the TEAD-YAP1 complex in RCCs. Because the prognosis of patients with high-grade ccRCC remains poor, it is an attractive hypothesis that unidentified molecules that are activated by suppressed Hippo signaling could be candidates for consideration in the development of new therapeutic drugs for high-grade ccRCCs. Further studies will be required to address this possibility.

## Conclusions

We show that SAV1, a component of the Hippo pathway, was frequently downregulated in high-grade ccRCC in association with copy number loss of chromosome 14q22.1, where the SAV1 gene is located. In addition to these studies using ccRCC samples, we examined the functional significance of SAV1 in cell lines. The results indicated that the downregulation of SAV1 promoted cell proliferation and inhibited apoptosis in ccRCC. In addition, YAP1 tended to be located in the nucleus in ccRCC cases with SAV1 downregulation, and it was preferentially detected in high-grade ccRCC. On the bases of these findings, downregulation of SAV1 and the consequent YAP1 activation are involved in the pathogenesis of high-grade ccRCC.

## Abbreviations

SAV1: Salvador homolog 1; ccRCC: Clear cell renal cell carcinoma; LATS: Large tumor suppressor; YAP: Yes-associated protein; TEAD TEA: Domain family member; CNA: Copy number aberration; RT-PCR: Real-time PCR; CGH: Comparative genomic hybridization; HIF: Hypoxia inducible factor; RPTEC: Renal proximal tubule epithelial cells; MST: Mammalian sterile STE20-like kinase; STK: Serine/threonine kinase; HCC: Hepatocellular carcinoma; EMT: Epithelial-mesenchymal transition

## Competing interests

The authors declare that they have no competing interests.

## Authors' contributions

KM and MM conceived the experiments; MS supervised the design of this work; TN, FS, and HM assisted in the acquisition of clinical material; MM, TN and MM assisted with collection of array data and analysis; MT and IT contributed to data validation; All authors were involved in writing the paper and had final approval of the submitted and published versions.

## Pre-publication history

The pre-publication history for this paper can be accessed here:

http://www.biomedcentral.com/1471-2407/11/523/prepub

## Supplementary Material

Additional file 1 **Table S1**. Information for ccRCC cases. Click here for file

Additional file 2 **Methods S1**. Methods. Click here for file

Additional file 3 **Figure S1**. Chromosomal imbalance in RCC cell lines and homozygous deletion. Click here for file

Additional file 4 **Figure S2**. Correlation of expression level with gene copy number for MAP4K5, SPG3A and HIF1a. Click here for file

Additional file 5 **Figure S3**. Immunohistochemistry of ccRCC tissue using anti-SAV1 antibody. Click here for file

Additional file 6 **Figure S4**. Immunocytochemistry of 786-O cells transduced with lentivirus expressing SAV1 or control virus using anti-SAV1 antibody. Click here for file

Additional file 7 **Figure S5**. Proliferation of 786-O and 769-P cells after re-expression of SAV1. Click here for file

Additional file 8 **Figure S6**. Cell cycle analysis of siRNA-trasnfected HK2cells. Click here for file

Additional file 9 **Figure S7**. Transcriptional activity of TEADs-YAP1 in SAV1-re-expressing clones. Click here for file

## References

[B1] Reuter VE The pathology of renal epithelial neoplasms Semin Oncol 2006 33 5 534 543 10.1053/j.seminoncol.2006.06.009 17045082

[B2] Fuhrman SA Lasky LC Limas C Prognostic significance of morphologic parameters in renal cell carcinoma Am J Surg Pathol 1982 6 7 655 663 10.1097/00000478-198210000-00007 7180965

[B3] Bretheau D Lechevallier E de Fromont M Sault MC Rampal M Coulange C Prognostic value of nuclear grade of renal cell carcinoma Cancer 1995 76 12 2543 2549 10.1002/1097-0142(19951215)76:12<2543::AID-CNCR2820761221>3.0.CO;2-S 8625083

[B4] Cambell SCNA Bukowski RM Wein AJ KL, Novick AC, Partin AW, Peters CA Renal tumors Cambell-Walsh UROLOGY 2007 9 Philadelphia: Saunders 1567 1637

[B5] Lane BR Babineau D Kattan MW Novick AC Gill IS Zhou M Weight CJ Campbell SC A preoperative prognostic nomogram for solid enhancing renal tumors 7 cm or less amenable to partial nephrectomy J Urol 2007 178 2 429 434 10.1016/j.juro.2007.03.106 17561141

[B6] Patard JJ Leray E Rioux-Leclercq N Cindolo L Ficarra V Zisman A De La Taille A Tostain J Artibani W Abbou CC Prognostic value of histologic subtypes in renal cell carcinoma: a multicenter experience J Clin Oncol 2005 23 12 2763 2771 15837991 10.1200/JCO.2005.07.055

[B7] Yoshimoto T Matsuura K Karnan S Tagawa H Nakada C Tanigawa M Tsukamoto Y Uchida T Kashima K Akizuki S High-resolution analysis of DNA copy number alterations and gene expression in renal clear cell carcinoma J Pathol 2007 213 4 392 401 10.1002/path.2239 17922474

[B8] Tapon N Harvey KF Bell DW Wahrer DC Schiripo TA Haber DA Hariharan IK salvador Promotes both cell cycle exit and apoptosis in Drosophila and is mutated in human cancer cell lines Cell 2002 110 4 467 478 10.1016/S0092-8674(02)00824-3 12202036

[B9] Matsuura K Uesugi N Hijiya N Uchida T Moriyama M Upregulated expression of cardiac ankyrin-repeated protein in renal podocytes is associated with proteinuria severity in lupus nephritis Hum Pathol 2007 38 3 410 419 10.1016/j.humpath.2006.09.006 17239933

[B10] Nakada C Matsuura K Tsukamoto Y Tanigawa M Yoshimoto T Narimatsu T Nguyen LT Hijiya N Uchida T Sato F Genome-wide microRNA expression profiling in renal cell carcinoma: significant down-regulation of miR-141 and miR-200c J Pathol 2008 216 4 418 427 10.1002/path.2437 18925646

[B11] Kosari F Parker AS Kube DM Lohse CM Leibovich BC Blute ML Cheville JC Vasmatzis G Clear cell renal cell carcinoma: gene expression analyses identify a potential signature for tumor aggressiveness Clin Cancer Res 2005 11 14 5128 5139 10.1158/1078-0432.CCR-05-0073 16033827

[B12] Shomori K Nagashima Y Kuroda N Honjo A Tsukamoto Y Tokuyasu N Maeta N Matsuura K Hijiya N Yano S ARPP protein is selectively expressed in renal oncocytoma, but rarely in renal cell carcinomas Mod Pathol 2007 20 2 199 207 10.1038/modpathol.3800730 17206105

[B13] Harvey K Tapon N The Salvador-Warts-Hippo pathway--an emerging tumour-suppressor network Nat Rev Cancer 2007 7 3 182 191 10.1038/nrc2070 17318211

[B14] Harvey KF Pfleger CM Hariharan IK The Drosophila Mst ortholog, hippo, restricts growth and cell proliferation and promotes apoptosis Cell 2003 114 4 457 467 10.1016/S0092-8674(03)00557-9 12941274

[B15] Wu S Huang J Dong J Pan D hippo encodes a Ste-20 family protein kinase that restricts cell proliferation and promotes apoptosis in conjunction with salvador and warts Cell 2003 114 4 445 456 10.1016/S0092-8674(03)00549-X 12941273

[B16] Zhao B Lei QY Guan KL The Hippo-YAP pathway: new connections between regulation of organ size and cancer Curr Opin Cell Biol 2008 20 6 638 646 10.1016/j.ceb.2008.10.001 18955139 PMC3296452

[B17] Zhao B Li L Lei Q Guan KL The Hippo-YAP pathway in organ size control and tumorigenesis: an updated version Genes Dev 2010 24 9 862 874 10.1101/gad.1909210 20439427 PMC2861185

[B18] Vassilev A Kaneko KJ Shu H Zhao Y DePamphilis ML TEAD/TEF transcription factors utilize the activation domain of YAP65, a Src/Yes-associated protein localized in the cytoplasm Genes Dev 2001 15 10 1229 1241 10.1101/gad.888601 11358867 PMC313800

[B19] Zhao B Ye X Yu J Li L Li W Li S Yu J Lin JD Wang CY Chinnaiyan AM TEAD mediates YAP-dependent gene induction and growth control Genes Dev 2008 22 14 1962 1971 10.1101/gad.1664408 18579750 PMC2492741

[B20] Kango-Singh M Singh A Regulation of organ size: Insights from the Drosophila Hippo signaling pathway Dev Dyn 2009 238 7 1627 1637 10.1002/dvdy.21996 19517570

[B21] Cai J Zhang N Zheng Y de Wilde RF Maitra A Pan D The Hippo signaling pathway restricts the oncogenic potential of an intestinal regeneration program Genes Dev 2010 24 21 2383 2388 10.1101/gad.1978810 21041407 PMC2964748

[B22] Pan D The hippo signaling pathway in development and cancer Dev Cell 2010 19 4 491 505 10.1016/j.devcel.2010.09.011 20951342 PMC3124840

[B23] Lee KP Lee JH Kim TS Kim TH Park HD Byun JS Kim MC Jeong WI Calvisi DF Kim JM The Hippo-Salvador pathway restrains hepatic oval cell proliferation, liver size, and liver tumorigenesis Proc Natl Acad Sci USA 2010 107 18 8248 8253 10.1073/pnas.0912203107 20404163 PMC2889558

[B24] Lu L Li Y Kim SM Bossuyt W Liu P Qiu Q Wang Y Halder G Finegold MJ Lee JS Hippo signaling is a potent in vivo growth and tumor suppressor pathway in the mammalian liver Proc Natl Acad Sci USA 2010 107 4 1437 1442 10.1073/pnas.0911427107 20080689 PMC2824398

[B25] Lee JH Kim TS Yang TH Koo BK Oh SP Lee KP Oh HJ Lee SH Kong YY Kim JM A crucial role of WW45 in developing epithelial tissues in the mouse Embo J 2008 27 8 1231 1242 10.1038/emboj.2008.63 18369314 PMC2367404

[B26] Dong J Feldmann G Huang J Wu S Zhang N Comerford SA Gayyed MF Anders RA Maitra A Pan D Elucidation of a universal size-control mechanism in Drosophila and mammals Cell 2007 130 6 1120 1133 10.1016/j.cell.2007.07.019 17889654 PMC2666353

[B27] Steinhardt AA Gayyed MF Klein AP Dong J Maitra A Pan D Montgomery EA Anders RA Expression of Yes-associated protein in common solid tumors Hum Pathol 2008 10.1016/j.humpath.2008.04.012 PMC2720436 18703216

[B28] Zender L Spector MS Xue W Flemming P Cordon-Cardo C Silke J Fan ST Luk JM Wigler M Hannon GJ Identification and validation of oncogenes in liver cancer using an integrative oncogenomic approach Cell 2006 125 7 1253 1267 10.1016/j.cell.2006.05.030 16814713 PMC3026384

[B29] Zhao B Wei X Li W Udan RS Yang Q Kim J Xie J Ikenoue T Yu J Li L Inactivation of YAP oncoprotein by the Hippo pathway is involved in cell contact inhibition and tissue growth control Genes Dev 2007 21 21 2747 2761 10.1101/gad.1602907 17974916 PMC2045129

[B30] Hall CA Wang R Miao J Oliva E Shen X Wheeler T Hilsenbeck SG Orsulic S Goode S Hippo pathway effector Yap is an ovarian cancer oncogene Cancer Res 2010 70 21 8517 8525 10.1158/0008-5472.CAN-10-1242 20947521 PMC2970655

