# Supplementary material for: Downregulation of SAV1 plays a role in pathogenesis of high-grade clear cell renal cell carcinoma
Source: BMC Cancer. 2011 Dec 20;11:523. doi: 10.1186/1471-2407-11-523 (PMC3292516; doi:10.1186/1471-2407-11-523)
Supplement: Additional file 1 — Table S1. Information for ccRCC cases. [file 1471-2407-11-523-S1.PDF]

Supplementary Table S1. Information for ccRCC cases

case	gender	age(years)	grade
**1**	m	74	3
**2**	m	79	3
**3**	f	73	3
**4**	f	55	2
**5**	f	73	2
**6**	m	61	3
**7**	f	75	1
**8**	f	61	3
**9**	f	79	2
**10**	m	76	3
**11**	m	50	3
**12**	m	47	3
**13**	f	71	3
**14**	m	39	3
**15**	f	65	3
**16**	m	60	3
**17**	f	49	3
**18**	f	42	2
**19**	m	55	3
**20**	f	76	3
**21**	m	75	3
**22**	m	75	4
**23**	m	68	3
**24**	m	62	3
**25**	m	70	3
**26**	f	34	1
**27**	m	54	2
**28**	m	60	4
**29**	m	65	3
**30**	f	58	3
**31**	m	54	2
**32**	m	73	4
**33**	m	47	2
**34**	m	80	4
**35**	f	66	2
**36**	m	57	3
**37**	f	70	2
**38**	m	79	1
**39**	m	81	3
**40**	m	74	3
**41**	m	77	4
**42**	m	69	2
**43**	m	55	3
**44**	m	74	3
**45**	f	78	2
**46**	m	75	3
**47**	f	35	3
**48**	m	64	3
**49**	f	76	3
**50**	m	64	3
**51**	m	34	3
**52**	m	62	2
**53**	f	84	2
**54**	m	59	3
**55**	m	85	2
**56**	m	57	3
**57**	f	72	3
**58**	m	76	3
**59**	f	63	2
**60**	m	43	3
**61**	f	68	2
**62**	f	58	3
**63**	m	57	3
**64**	m	60	3
**65**	m	76	3
**66**	f	76	2
**67**	f	56	1
**68**	m	70	3
**69**	m	75	2
**70**	f	63	3
**71**	m	64	3
**72**	m	51	3
**73**	m	79	2
**74**	f	59	3
**75**	m	53	3
**76**	m	77	2
**77**	m	87	3
**78**	m	48	3
**79**	m	54	3
**80**	m	40	2
**81**	m	67	3
**82**	f	36	3
**83**	m	72	4
**84**	m	59	2
**85**	m	54	3
**86**	m	74	4
**87**	m	58	4
**88**	m	65	3
**89**	m	65	2
**90**	m	48	2
**91**	m	79	3
**92**	m	74	1
**93**	m	78	3
**94**	m	36	3
**95**	m	75	3
**96**	f	69	3
**98**	m	65	4
**99**	m	58	4

m; male, f; female
