# Supplementary material for: Downregulation of SAV1 plays a role in pathogenesis of high-grade clear cell renal cell carcinoma
Source: BMC Cancer. 2011 Dec 20;11:523. doi: 10.1186/1471-2407-11-523 (PMC3292516; doi:10.1186/1471-2407-11-523)
Supplement: Additional file 2 — Methods S1. Methods. [file 1471-2407-11-523-S2.PDF]

Supplementary Methods

DNA extraction

Genomic DNA was extracted from cells in accordance with the standard proteinase K digestion method, followed by phenol/chloroform extraction.

Array CGH

The Agilent Human Genome CGH microarray 44K (Agilent Technologies, Inc., Palo Alto, CA, USA) was used for array-CGH experiments. Genomic DNA from cell lines and from eight healthy male donors (each sample 2 μg) was subjected to array-CGH in accordance with the manufacturer's instructions. The arrays were washed in accordance with the manufacturer's recommendations, scanned with an Agilent 2565AA DNA microarray scanner (Agilent Technologies), and processed with Agilent Feature Extraction software (version 6.1.1, Agilent Technologies). All the data obtained in the aCGH analysis are available at DDBJ via CIBEX (cibex) under accession numbers for the array design, CBX97. All the data obtained from microarray analysis using primary ccRCCs are available at DDBJ via CIBEX (cibex) under accession numbers for the array design, CBX24. Use of the tissue samples for all experiments was approved by all the patients concerned, and by Oita University Ethics Committee (Approval No. P-05-05).

RNA extraction and microarray experiments

Total RNA extraction, analysis for quantity and quality, and microarray experiments were

performed as reported previously. All the data obtained in the expression-microarray analysis are available at DDBJ via CIBEX (cibex) under the array design accession number CBX96.

Immunocytochemistry

Cells were fixed with 4% paraformaldehyde for 20 min at room temperature. After washing with PBS, cells were permeabilized in 0.3% Triton X-100/PBS for 15 min at room temperature. The cells were then preincubated in normal rabbit serum, followed by incubation with anti-SAV1 antibody as a first antibody, and anti-mouse IgG as a second antibody.

Cell cycle analysis

The cells were harvested with trypsin-EDTA after transfection for 48 h, washed with PBS twice, and fixed in 70% ethanol at -20°C overnight. The fixed cells were pelleted and incubated in PBS containing 50 μg/ml propidium iodide (PI) and 5 mg/ml RNase A at 37°C for 30 min. Analysis of DNA content was carried out in a FACScan Flow Cytometer using CellQuest software for acquisition (BD Biosciences) and ModFit software for analysis (Verity Software House, Topsham, ME). Ten thousand events were analyzed per sample.

Proliferation assay

After transfection with siRNAs in a 96-well plate, MTS assay was carried out using the CellTiter 96 Aqueous cell proliferation assay (Promega, Madison, WI, USA), and the

optical density was measured at 492 nm using a fluorescence reader (Tecan SpectraFluor) (Tecan, Crailsheim, Germany).

Dual luciferase assay

For the luciferase reporter assay, stable clones of 786-O cells were seeded in 6-well plates. Luciferase reporter, GAL4-TEADs, and pGL4.35(9 xUAS Gal4) were co-transfected using Lipofectamine Plus (Invitrogen). At 48 hours after transfection, the cells were lysed and luciferase activity was assayed using a Dual-luciferase Reporter Assay kit (Promega, Madison, WI) in accordance with he manufacturer's instructions. All luciferase activities were normalized to the *Renilla* luciferase reporter pRL-CMV plasmid (Promega).

Statistical analysis

Quantitative RT-PCR data were analyzed statistically by the Mann-Whitney U-test. The two-sided Student's *t* test was used to analyze differences in experimental data obtained from the cell lines and ccRCC cases. P values of <0.05 were considered statistically significant.

References

1. Zhao B, Ye X, Yu J*, et al.* TEAD mediates YAP-dependent gene induction and growth control. *Genes Dev* 2008; **22**: 1962-1971.
