# Supplementary material for: Downregulation of SAV1 plays a role in pathogenesis of high-grade clear cell renal cell carcinoma
Source: BMC Cancer. 2011 Dec 20;11:523. doi: 10.1186/1471-2407-11-523 (PMC3292516; doi:10.1186/1471-2407-11-523)
Supplement: Additional file 3 — Figure S1. Chromosomal imbalance in RCC cell lines and homozygous deletion. [file 1471-2407-11-523-S3.PDF]

Supplementary Figure S1

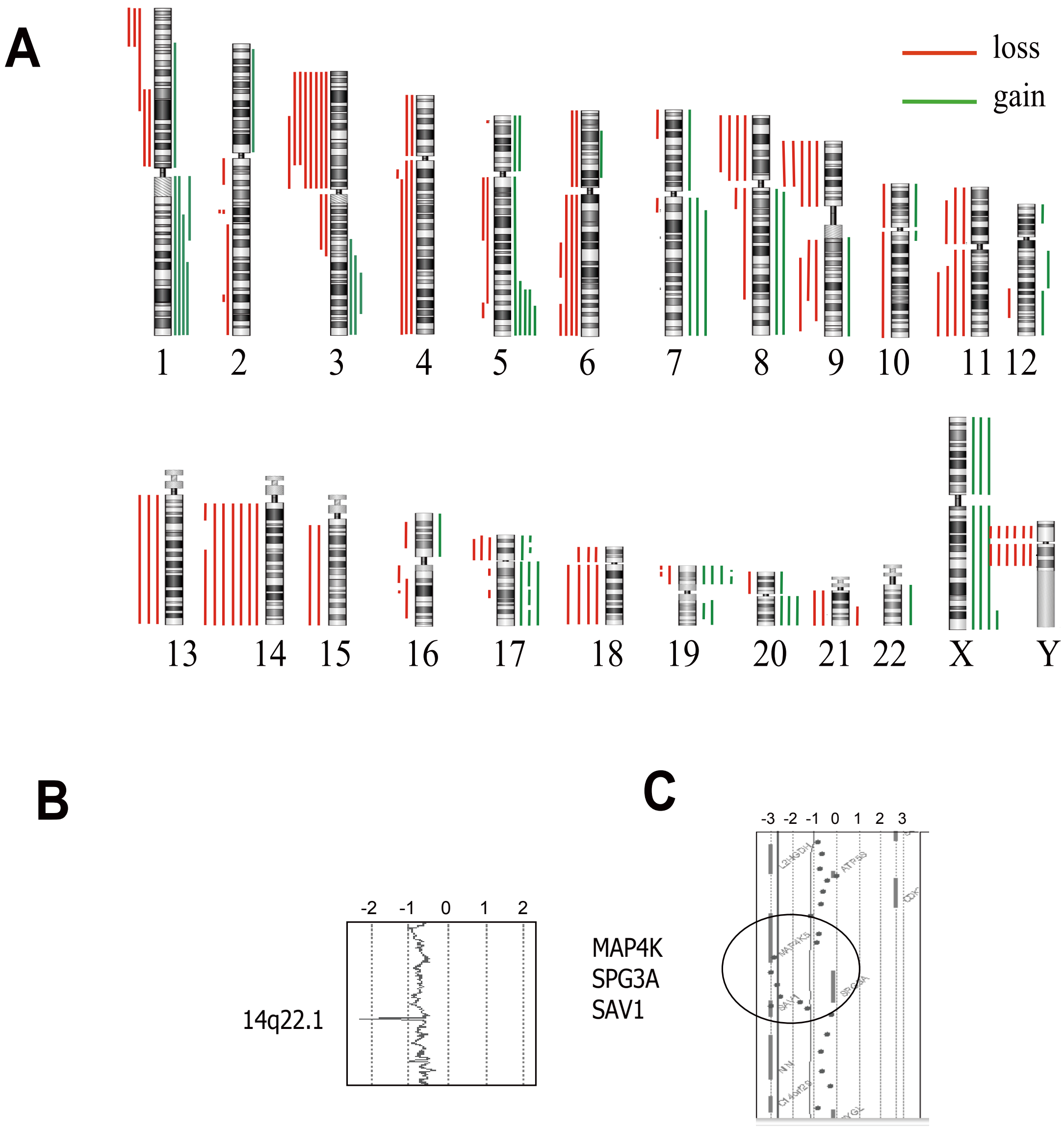

Supplementary Figure S1: Chromosomal imbalance in RCC cell lines and homozygous deletion

A, Summary of chromosomal imbalance in 8 RCC cell lines (786-O, 769-P, KMRC-1, KMRC-2, KMRC-3, KMRC-20, TUHR4TKB, and Caki-2). Lines on the left (red) of the ideogram indicate losses, and those on the right (green) indicate gains. **B,** Homozygous deletion at 14q22.1 in 786-O cells. Log_2 ratios (vertical dotted lines) are labeled on the top as -2, -1, 0, 1 and 2, with 0 as the baseline. **C;** homozygous deletion at the locus of *MAP4K*, *SPG3A* and *SAV1*. A single dot shows the signal intensity of each oligonucleotide probe, and the log_2 ratios are labeled on the top as -3, -2, -1, 0, 1, 2 and 3, with 0 as the baseline. Broad vertical lines indicate the locations of genes.
