# Supplementary material for: Downregulation of SAV1 plays a role in pathogenesis of high-grade clear cell renal cell carcinoma
Source: BMC Cancer. 2011 Dec 20;11:523. doi: 10.1186/1471-2407-11-523 (PMC3292516; doi:10.1186/1471-2407-11-523)
Supplement: Additional file 4 — Figure S2. Correlation of expression level with gene copy number for MAP4K5, SPG3A and HIF1a. [file 1471-2407-11-523-S4.PDF]

Supplementary Figure S2

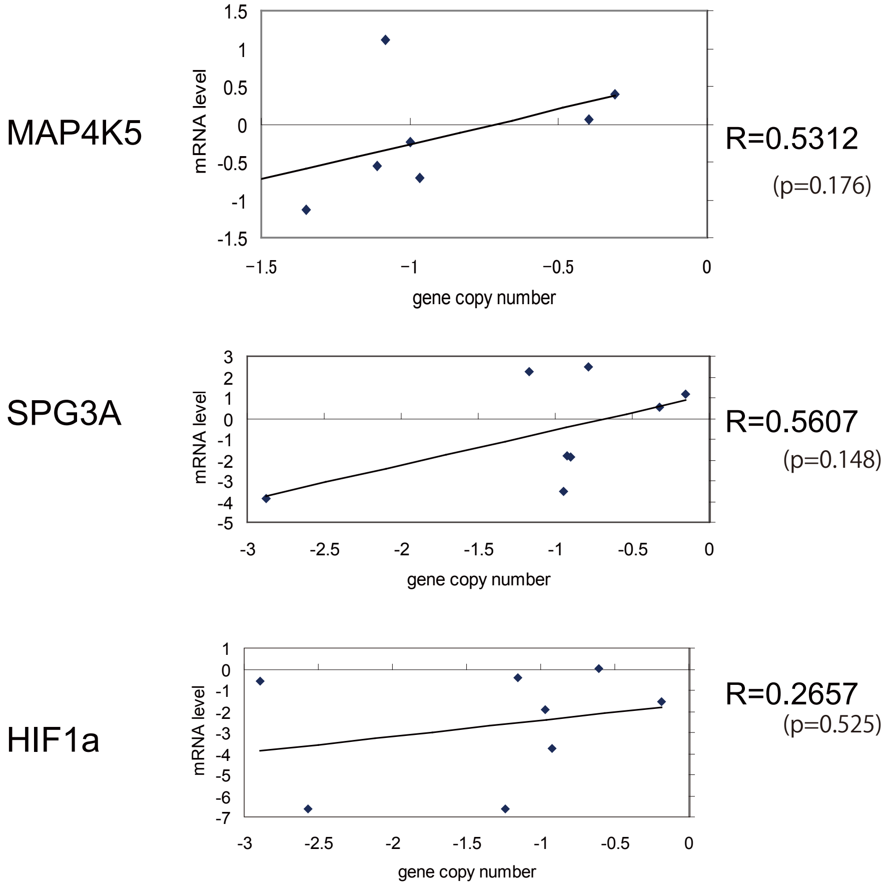

Supplementary Figure S2: Correlation of expression level with gene copy number for MAP4K5, SPG3A and HIF1a

Correlation of the copy number of the MAP4K5, SPG3A and HIF1a genes with the corresponding expression ratios in 8 RCC cell lines. Log_2 ratios of the normalized relative signal intensity shown by microarray (Y-axis) and the log_2 ratios of the gene copy number (X-axis) are plotted for each cell line. R, Pearson correlation coefficient. p-values were calculated by *t* test.
