# Supplementary material for: Downregulation of SAV1 plays a role in pathogenesis of high-grade clear cell renal cell carcinoma
Source: BMC Cancer. 2011 Dec 20;11:523. doi: 10.1186/1471-2407-11-523 (PMC3292516; doi:10.1186/1471-2407-11-523)
Supplement: Additional file 5 — Figure S3. Immunohistochemistry of ccRCC tissue using anti-SAV1 antibody. [file 1471-2407-11-523-S5.PDF]

Supplementary Figure S3

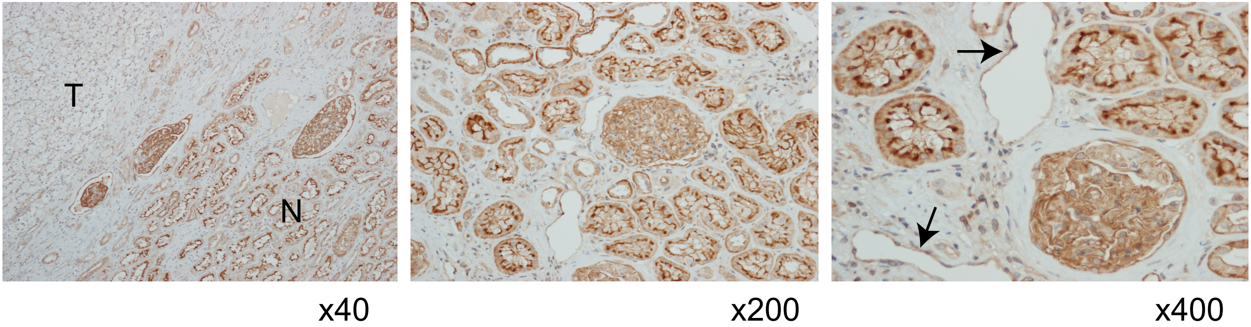

Supplementary Figure S3: Immunohistochemistry of ccRCC tissue using anti-SAV1 antibody

Representative result of immunohistochemistry using anti-SAV1 antibody is shown. SAV1 protein is downregulated in tumor cells, whereas it is strongly expressed in proximal renal tubules, podocytes, and endothelial cells in normal tissues. Left column; x40, N; normal tussues, T; tumor cells. Middle column; x200, Right column; x400, arrows indicate endothelial cells.
