# Supplementary material for: Downregulation of SAV1 plays a role in pathogenesis of high-grade clear cell renal cell carcinoma
Source: BMC Cancer. 2011 Dec 20;11:523. doi: 10.1186/1471-2407-11-523 (PMC3292516; doi:10.1186/1471-2407-11-523)
Supplement: Additional file 6 — Figure S4. Immunocytochemistry of 786-O cells transduced with lentivirus expressing SAV1 or control virus using anti-SAV1 antibody. [file 1471-2407-11-523-S6.PDF]

Supplementary Figure S4

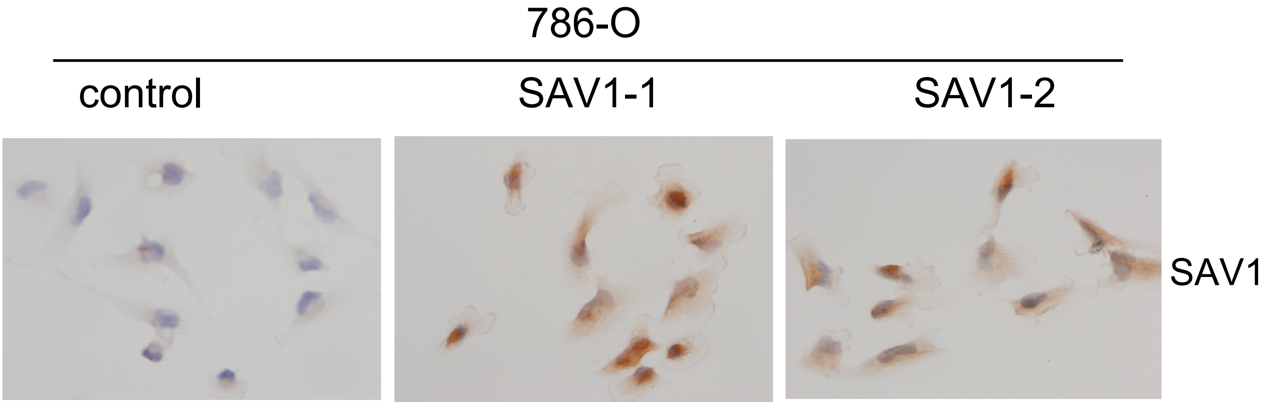

Supplementary Figure S4: Immunocytochemistry of 786-O cells transduced with lentivirus expressing SAV1 or control virus using anti-SAV1 antibody

786-O cells were transduced with pLenti6.3/V5-DEST empty vector or SAV1-pLenti6.3/V5-DEST, and 2 cell lines that express SAV1 (SAV1-1 and SAV1-2) and a control cell line that were transduced with empty vector alone (control) were established. Immunocytochemistry of these cells with anti-SAV1 antibody evidently shows that the antibody is specifically immunoreactive with SAV1.
