# Supplementary material for: Downregulation of SAV1 plays a role in pathogenesis of high-grade clear cell renal cell carcinoma
Source: BMC Cancer. 2011 Dec 20;11:523. doi: 10.1186/1471-2407-11-523 (PMC3292516; doi:10.1186/1471-2407-11-523)
Supplement: Additional file 7 — Figure S5. Proliferation of 786-O and 769-P cells after re-expression of SAV1. [file 1471-2407-11-523-S7.PDF]

Supplementary Figure S5

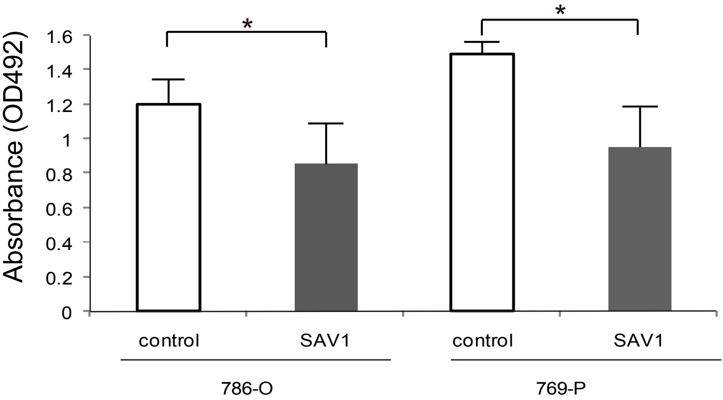

Supplementary Figure S5: Proliferation of 786-O and 769-P cells after re-expression of SAV1

Re-expression of SAV1 inhibits proliferation of 786-O and 769-P cells. 786-O and 769-P cells transduced with the pLenti7.3/V5 empty vector (control) or with SAV1- pLenti7.3/V (SAV1) were analyzed by MTS assay at 72 h after transfection. Y axis indicates the absorbance at 492 nm for MTS assay. Experiments were performed in triplicate, and bars indicate s.d.. * p <0.001; Student's *t* test.
