# Supplementary material for: Downregulation of SAV1 plays a role in pathogenesis of high-grade clear cell renal cell carcinoma
Source: BMC Cancer. 2011 Dec 20;11:523. doi: 10.1186/1471-2407-11-523 (PMC3292516; doi:10.1186/1471-2407-11-523)
Supplement: Additional file 8 — Figure S6. Cell cycle analysis of siRNA-trasnfected HK2cells. [file 1471-2407-11-523-S8.PDF]

Supplementary Figure S6

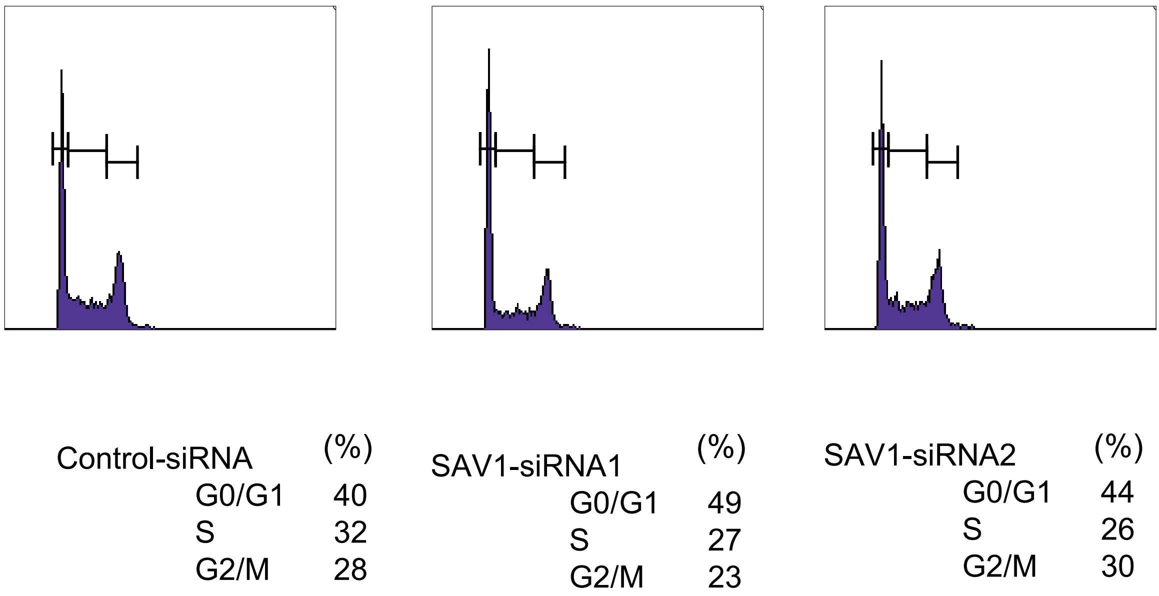

Supplementary Figure S6: Cell cycle analysis of siRNA-trasnfected HK2cells

Flow cytometry analysis of the DNA content of siRNA-transfected HK2 cells using PI staining. Two independent Stealth^{TM} RNAi oligonucleotides for each SAV1 (SAV1-siRNA1 and SAV1-siRNA2), or the Stealth^{TM} RNAi Negative Control Duplex siRNAs (control-siRNA) were transfected into HK2 cells, and the cells were harvested 72 h later. The relative percentages of cells in the G0/G1, S, and G2/M phases are shown.
