# Supplementary material for: Downregulation of SAV1 plays a role in pathogenesis of high-grade clear cell renal cell carcinoma
Source: BMC Cancer. 2011 Dec 20;11:523. doi: 10.1186/1471-2407-11-523 (PMC3292516; doi:10.1186/1471-2407-11-523)
Supplement: Additional file 9 — Figure S7. Transcriptional activity of TEADs-YAP1 in SAV1-re-expressing clones. [file 1471-2407-11-523-S9.PDF]

Supplementary Figure S7

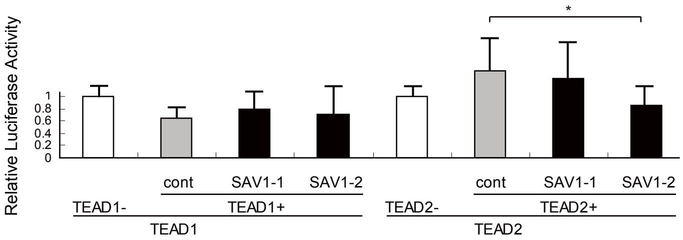

Supplementary Figure S7: Transcriptional activity of TEADs-YAP1 in SAV1-re-expressing clones

SAV1 suppressed the transcriptional activity of TEADs-YAP1 in a TEAD-dependent manner. SAV1-1, SAV1-2 and control cells (stable clones established by transduction with SAV1-pLenti6.3/V5-DEST or pLenti6.3/V5-DEST empty vector in 786-O cells) were transfected with TEAD1 (Gal4-TEAD1) or TEAD2 (Gal4-TEAD2), together with a Gal4-9x UAS luciferase reporter (pGL4.31) and pRL-CMV. Gal4-TEAD1 and Gal4-TEAD2 plasmid vectors that expressed TEAD1 or TEAD2 fused to GAL4 were used (kindly provided by Dr. B. Zhao [1]). In SAV1-1 and SAV1-2 cells, the luciferase activity was not changed by co-transfection with Gal4-TEAD1, but was slightly decreased when Gal4-TEAD2 was co-transfected. Firefly luciferase activity was normalized to Renilla luciferase activity. Normalized luciferase activity in TEAD1-untrasfected control cells was set at 1. Experiments were performed in triplicate. The dual luciferase data are shown as mean ± SD. *$p<0.05$ *; Student's *t* test.
